# Double-strand breaks in lymphocyte DNA of humans exposed to [^18^F]fluorodeoxyglucose and the static magnetic field in PET/MRI

**DOI:** 10.1186/s13550-020-00625-1

**Published:** 2020-04-28

**Authors:** Gunnar Brix, Elisabeth Günther, Ute Rössler, David Endesfelder, Alexandra Kamp, Ambros Beer, Matthias Eiber

**Affiliations:** 1 Department of Medical and Occupational Radiation Protection, Federal Office for Radiation Protection, Neuherberg, Germany; 2 Department of Nuclear Medicine, Technical University of Munich, Munich, Germany; 3 Department of Effects and Risks of Ionizing and Non-Ionizing Radiation, Federal Office for Radiation Protection, Neuherberg, Germany; 4 Department of Nuclear Medicine, University Ulm, Ulm, Germany

**Keywords:** PET/MRI, Static magnetic field, [^18^F]fluorodeoxyglucose, Genotoxicity, γH2AX assay, Synergistic effects

## Abstract

**Background:**

Given the increasing clinical use of PET/MRI, potential risks to patients from simultaneous exposure to ionising radiation and (electro)magnetic fields should be thoroughly investigated as a precaution. With this aim, the genotoxic potential of 2-deoxy-2-[^18^F]fluoro-D-glucose ([^18^F]FDG) and a strong static magnetic field (SMF) were evaluated both in isolation and in combination using the γH2AX assay detecting double-strand breaks in lymphocyte DNA.

**Methods:**

Thirty-two healthy young volunteers allocated to three study arms were exposed to [^18^F]FDG alone, to a 3-T SMF alone or to both combined over 60 min at a PET/CT or a PET/MRI system. Blood samples taken after in vivo exposure were incubated up to 60 min to extend the irradiation of blood by residual [^18^F]FDG within the samples and the time to monitor the γH2AX response. Absorbed doses to lymphocytes delivered in vivo and in vitro were estimated individually for each volunteer exposed to [^18^F]FDG. γH2AX foci were scored automatically by immunofluorescence microscopy.

**Results:**

Absorbed doses to lymphocytes exposed over 60 to 120 min to [^18^F]FDG varied between 1.5 and 3.3 mGy. In this time interval, the radiotracer caused a significant median relative increase of 28% in the rate of lymphocytes with at least one γH2AX focus relative to the background rate (*p* = 0.01), but not the SMF alone (*p* = 0.47). Simultaneous application of both agents did not result in a significant synergistic or antagonistic outcome (*p* = 0.91).

**Conclusion:**

There is no evidence of a synergism between [^18^F]FDG and the SMF that may be of relevance for risk assessment of PET/MRI.

## Introduction

Due to impressing technological advances achieved in recent years [1–3], hybrid PET/MRI has become an established imaging procedure in clinical practice. Despite of an ongoing debate about specific clinical indications, it undoubtedly offers promising potential for personalised medicine based on anatomic, functional and molecular image data acquired in a single examination [4–7]. Furthermore, PET/MRI avoids exposure to X-rays substantially contributing to the exposure of patients in PET/CT or SPECT/CT [8, 9]. On the other hand, there are also risks and health hazards to patients associated with the three different (electro)magnetic fields used in MRI [10] so that exposure levels are limited [11].

Current regulations and safety standards, however, address only risks related either to ionising radiation or (electro)magnetic fields without considering potential synergisms (or antagonisms) between them – such as on DNA damage and repair that may result in an increased genotoxicity of PET radiotracers [12]. Increasing clinical uses of PET/MRI therefore require to thoroughly investigate this aspect at exposure levels occurring at PET/MRI systems.

Of particular interest are potential synergisms between the strong uniform static magnetic field (SMF) used in MRI and ionising radiation emitted by PET tracers. Patients undergoing PET/MRI examinations are continuously exposed to both agents over a longer period. Moreover, various cell and animal studies found that SMF in combination with ionising radiation produces a significantly different outcome compared with ionising radiation alone [13]. Since synergisms were observed even when the SMF was applied before or after irradiation, changes of the dose distribution by SMFs can likely be excluded as a relevant interaction mechanism [13], as supported by Monte Carlo simulations [14, 15]. A recent review on combined effects of the two agents [13] concluded that the current evidence is insufficient to identify the underlying mechanism(s) of the observed synergisms. The authors suppose that SMF may (i) modify one or more steps in the DNA damage response, (ii) influence the yield or lifetime of reactive oxygen species responsible for indirect DNA damage, or (iii) influence intracellular signalling implicated in non-targeted radiation-induced effects.

To monitor DNA double-strand breaks (DSBs), the γH2AX assay has been established as a sensitive and direct molecular marker that has been used to investigate the genotoxic potential of CT, PET, and MRI on humans (e.g. [16–24]). The state of science can be briefly summarised as follows [25–28]: H2AX is an ubiquitous appearing DNA-associated histone that is involved in the structural organisation of the DNA, particularly in the repair of DSBs. Flanking each site of a DSB, a large number of H2AX histones become phosphorylated. The resulting foci of phosphorylated H2AX histones (referred to as γH2AX) can be detected microscopically after immunofluorescence staining. Due to this amplification process, radiation-induced DSBs can be monitored even at very low dose levels down to 1 mGy [25]. Once a lesion is repaired, γH2AX is dephosphorylated and no longer visible. Formation of γH2AX foci is observed most commonly in peripheral lymphocytes (re)distributed in blood flow and thus being representative of exposure of all organs and tissues.

The present prospective study was aimed at investigating the genotoxicity of 2-deoxy-2-[^18^F]fluoro-D-glucose ([^18^F]FDG) and the SMF used in MRI alone and combined by the γH2AX assay.

## Materials and methods

### Compliance with ethical standards

The prospective study was approved by the institutional review board as well as the competent federal radiation protection authority and was performed in accordance with the ethical standards laid down in the Declaration of Helsinki. Informed consent was obtained individually from all participants included in the study.

### Volunteers

The study was designed as a prospective, three-armed trial with volunteers exposed to [^18^F]FDG alone (study arm SA1), the SMF alone (SA2), and both agents combined (SA3). The null hypothesis was that there is no difference between the study arms. To obtain a power of 80% (Kruskal-Wallis test), given an effect greater than 1.5-fold of the inter-individual variance within the study arms, a minimum of 10 participants had to be investigated per arm.

Included were younger individuals not diagnosed with any disease that may alter DNA damage and repair [29]. Contraindications were an existing pregnancy, blood glucose concentrations of less than 80 or more than 180 mg/dl in case of an exposure to [^18^F]FDG, as well as active or passive implants or other objects of ferromagnetic or unknown material in case of an exposure to the SMF. The volunteers received a payment depending on the study arm [29].

### Exposure scenarios, blood sampling, and incubation

**Fig. 1:**
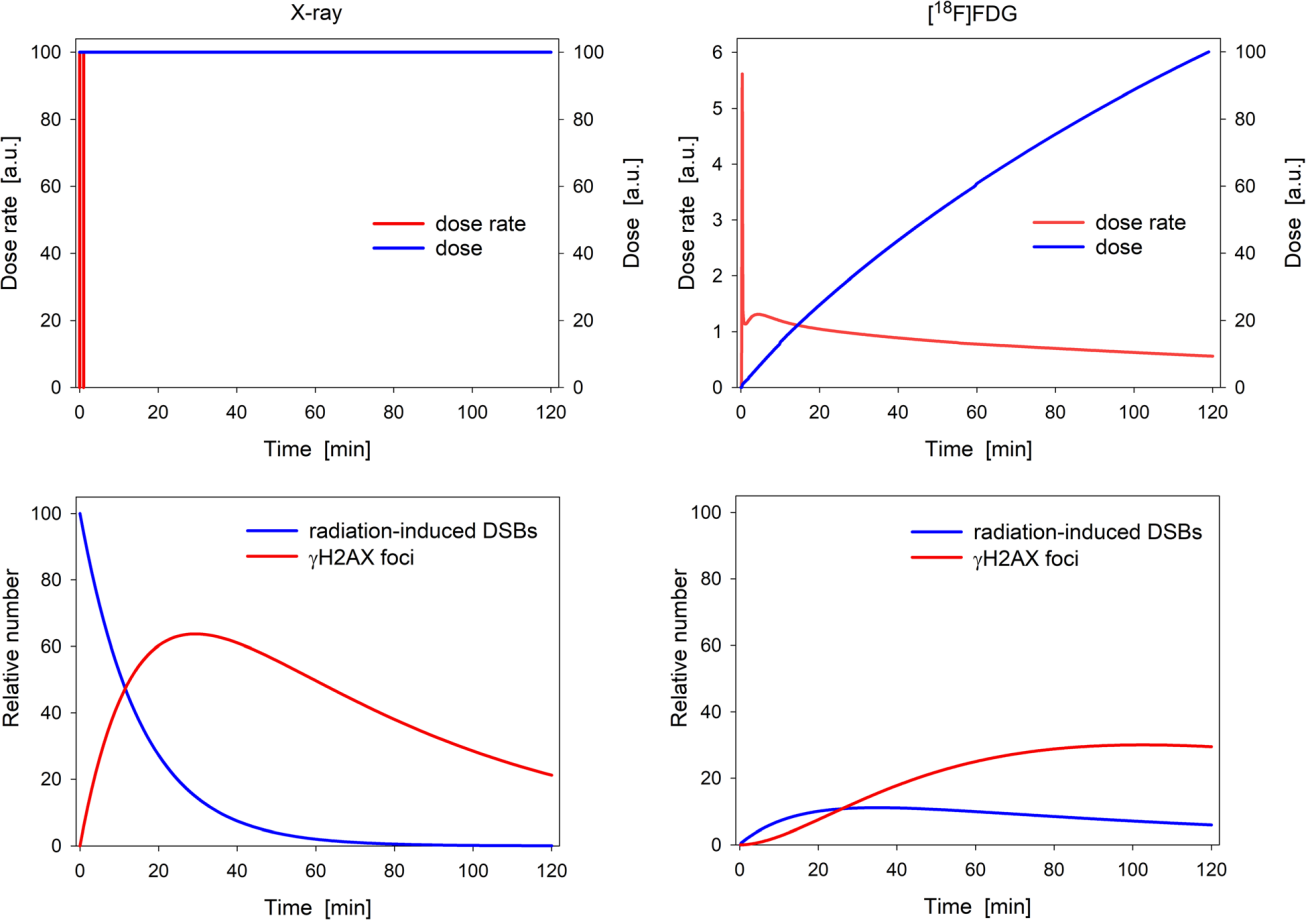
Schematic representation of the differences between X-ray (left column) and radiotracer (right column) exposure on the formation and repair of DSBs monitored by the γH2AX assay. The upper row compares the dose rate and the resulting absorbed dose to blood following a quasi-instantaneous X-ray exposure and [^18^F]FDG administration (real data of a volunteer) assuming that the absorbed dose to lymphocytes is identical at the end of the considered time range. In the lower row, simulated kinetics of DSBs not yet under repair and of γH2AX foci are plotted assuming that the maximum of foci rates occurs 30 min after X-ray exposure [17, 26, 27] and that the rate constants characterizing the formation and dephosphorylation of γH2AX foci are comparable for both exposure scenarios. Prolonged exposure to [^18^F]FDG results in a markedly delayed and flattened γH2AX response at an overall lower level

**Fig. 2:**
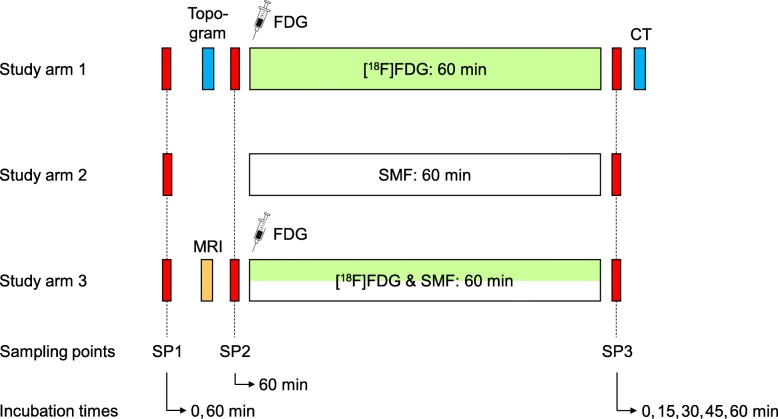
Design of the three-armed study indicating the investigated exposure scenarios as well as the blood sampling and incubation schemes

As schematically shown in Fig. 1, the γH2AX response to a prolonged exposure of lymphocytes to [^18^F]FDG is markedly delayed compared to a quasi-instantaneous X-ray exposure and should thus be observed over a longer period. On the other hand, the time the volunteers had to lie still in the imaging systems to acquire activity concentration-time courses for dosimetry had to be limited. To balance these aspects, volunteers were exposed in vivo over 60 min. Nevertheless, in order to extend the duration of blood exposure to [^18^F]FDG as well as the time for the formation of exposure-induced γH2AX foci, blood was taken at the end of this period (sampling point SP3) and incubated in vitro up to 60 min, before lymphocytes were fixed. To determine the background of γH2AX foci, blood was also collected prior to the exposure phase (SP1, SP2). For each of the three study arms, the examination protocol was adapted as far as necessary to the respective exposure and measurement conditions, as illustrated by the flow charts in Fig. 2. Details are described in the following.

Volunteers were exposed to [^18^F]FDG alone (SA1) at a PET/CT system (Biograph mCT; Siemens Healthineers, Erlangen, Germany) or to a 3-T SMF alone (SA2) and in combination with the radiotracer (SA3) at a PET/MRI system (Biograph mMR; Siemens). Technical details of the systems can be found in [30].

#### Study arm 1

Following the acquisition of an X-ray topogram (tube voltage, 120 kV; tube current, 25 mAs) and administration of a body weight-adapted [^18^F]FDG activity (nominal, 4.3 MBq/kg) through an antecubital vein, emission data were detected in the list mode over 60 min from an abdominal body region. For quantitative evaluation of activity concentrations, attenuation-corrected PET images were reconstructed using low-dose CT data acquired after blood sampling at SP3 from the same body region. This time sequence avoids a substantial additional exposure of lymphocytes taken at SP3 to X-rays that would otherwise interfere with the effects of [^18^F]FDG and the SMF to be investigated. PET images were reconstructed for 55 non-equidistant time frames. For dosimetry, the average [^18^F]FDG activity concentration in blood was determined for each time frame by evaluating small circular regions of interest placed over the centre of the aorta on 11 transversal PET images in the middle of the axial scan region. Blood was collected before (SP1) and after the topogram (SP2) as well as 60 min post [^18^F]FDG injection (SP3).

#### Study arm 2

Blood was withdrawn from volunteers before (SP1) and after (SP3) exposure to the 3-T SMF of the PET/MRI system.

#### Study arm 3

MR images required for transmission correction of emission data were acquired for technical reasons at the beginning of the examination procedure using a T1-weighted Dixon VIBE sequence (specific absorption rate, < 2 W/kg). Apart from that, blood sampling as well as data and image postprocessing were identical to study arm 1.

Blood was collected from the antecubital vein contralateral to the site of [^18^F]FDG administration via an 18- or 20-gauge indwelling cannula (Vasofix Safety IV; B. Braun Melsungen AG, Melsungen, Germany) into 7.5-ml lithium heparin monovettes (S-Monovette; Sarstedt AG & Co, Nümbrecht, Germany). Two monovettes were filled at SP1 and one at SP2. At SP3, blood was sequentially withdrawn into three monovettes, mixed together in 50-ml tubes (Falcon; Fisher Scientific GmbH, Schwerte, Germany) and then immediately portioned into five 15-ml centrifuge tubes (Falcon; Fisher Scientific GmbH). By this procedure, variations amongst sequentially collected blood were avoided as a source of error.

Blood samples were incubated in part at 37 ^°^C over different periods as indicated in Fig. 2. Pre-exposure samples taken at SP1 were incubated over 0 and 60 min to detect and—if present—consider potential distortive effects of venipuncture and incubation on data analysis. Due to a relevant time delay between the potential induction of DSBs by the X-ray topogram (SA1) or MRI scan (SA3) and the formation of detectable γH2AX foci, blood samples withdrawn at SP2 were incubated over 60 min. To extend the exposure of lymphocytes to residual [^18^F]FDG activity in the samples and to assess a potential continuing influence of the SMF on the genotoxicity of [^18^F]FDG beyond the end of field exposure, samples taken at SP3 were incubated over 0, 15, 30, 45, and 60 min.

Hereinafter, the biological condition of lymphocyte DNA isolated from blood samples is characterised by the study arm (SA), the blood sampling point (SP), and the incubation time (IT).

### Internal dosimetry

To estimate the total absorbed dose to blood as a surrogate for the dose to circulating lymphocytes, two contributions were considered: (i) the dose delivered in vivo to blood during the first 60 min p.i. by self-irradiation as well as cross-fire irradiation from other body tissues. (ii) The additional dose delivered subsequently in vitro during incubation, due to self-irradiation from the residual [^18^F]FDG activity in the samples.

#### In vivo exposure

The time-course of [^18^F]FDG activity in blood was estimated from measured activity concentration-time courses, using blood volumes determined from the body weight and height of the volunteers [31]. Both the self and cross-fire contributions to the total blood dose were estimated according to the MIRD approach [32]. In contrast to blood, the activities in other body tissues were not available for the volunteers and hence were estimated by using the model of Hays and Segall [33]. The required specific absorbed fractions (SAFs) were computed for the adult ICRP male and female reference voxel phantoms [34] and monoenergetic positron sources with the Monte Carlo code EGSnrc [35, 36]. Finally, the resulting monoenergetic data were convoluted with the β^+^-spectrum of ^18^F [37].

#### In vitro exposure

The exact geometries of the used 15-ml conical centrifuge tubes containing different predefined blood volumes were created using commercial software (Rhinoceros V5.0; Robert McNeel & Associates, Seattle, USA) in polygon-mesh format and voxelised. SAFs for the digitalised geometries were determined by Monte Carlo computations. For blood volumes deviating from the five predefined values, SAFs were estimated via a logarithmic interpolation of the simulated data. Doses delivered in vitro to the samples over incubation periods of 15, 30, 45, or 60 min were computed using the volunteer-specific residual activity concentrations in the aorta 60 min p.i as initial value.

### Processing of blood specimen and γH2AX assay

Following incubation, blood samples were kept at 5 °C until isolation of peripheral blood leucocytes. This was performed by density gradient centrifugation (10 min, 1000g, 5 °C) using 12-ml separation tubes (Leucosep Tube; Greiner Bio-One GmbH, Frickenhausen, Germany) and separation medium (Histopaque-1077; Sigma Aldrich Chemie GmbH, Taufkirchen, Germany). After centrifugation, leucocytes were visible and transferred into 5 ml cell culture medium (RPMI 1640; Pan-Biotech GmbH, Aidenbach, Germany). Cell suspension was centrifuged again (10 min, 250g, 5 °C), the medium decanted, and cells fixed in 1 ml 2% paraformaldehyde (PFA; Sigma Aldrich)/phosphate buffered saline (Dulbecco’s PBS; Biochrom GmbH, Berlin, Germany) solution for 15 min at 5 °C before centrifugation (10 min, 250g, 5 °C). After decanting the solution, lymphocytes were stained with Tuerk’s solution (Merck, Darmstadt, Germany), counted in a counting chamber and concentrated to one million cells per ml in PBS. Cell suspensions were stored at 5 °C.

One hundred microliters of cell suspension was spotted onto glass slides by cytospin centrifugation for 5 min at 54*g*. Slides were washed three times in fresh PBS containing 0.15% of a nonionic surfactant (TritonX-100; Sigma Aldrich) each time for 5 min, followed by three washing steps in blocking solution (1 g bovine serum albumin (BSA; Sigma Aldrich) mixed with 0.15 g glycine (Sigma Aldrich) in 100 ml PBS) each for 10 min. Seventy-five microliters blocking solution with anti-phosphohistone H2A.X (Ser139) rabbit mAb (Cell Signaling Technology Europe B.V., Frankfurt a.M., Germany) in the dilution 1:200 was transferred on each slide and incubated at 4 °C for at least 16 h. Slides were washed again for 5 min in PBS, for 10 min in PBS/Triton and for 5 min in PBS. Before incubating with the secondary antibody, an anti-rabbit IgG (H+L), F(ab')_2_ fragment conjugated to Alexa Fluor 555 fluorescent dye (Cell Signaling Technology Europe), in the dilution 1:1000 in blocking solution in a humid chamber for 45 min at room temperature, slides were treated with blocking solution for 7 min. After antibody binding, slides were washed twice in PBS/Triton for 5 min each, once in PBS for 10 min and once in PBS for 7 min. Cell nuclei were counterstained with Hoechst 33342 (Bisbenzimide H 33342 trihydrochloride; Sigma Aldrich) for 2 min and slides were washed again in PBS for 2 min twice. Finally, slides were covered by 16 μl antifade mounting medium (Vectashield; Vector Laboratories Inc., Burlingame, USA).

Search and image acquisition of cell nuclei on the slides was performed by automatic fluorescence microscopy using a scanning and imaging platform (Metafer 4, version V3.13.1; MetaSystems Hard & Software GmbH, Altlussheim, Germany) equipped with an objective (ZeissPlan-Neofluar 40×/0.75; Carl Zeiss Microscopy GmbH, Jena, Germany) yielding a 400-fold magnification. For foci analysis a Spectrum Orange bandpass filter (excitation: centre wavelength/bandwidth = 546/10 nm, emission 580/30 nm; Chroma 31003; Chroma Technology, Olching, Germany) and for counterstaining a DAPI bandpass filter (excitation 350/50 nm, emission 460/50 nm; Chroma 31000; Chroma Technology) was used. The threshold for foci intensity was set to 70% with a maximum gain of 500%. For lymphocytes exposed to very low doses and controls, cells with a high number of foci are not to be expected. Therefore, cells with more than five detected ‘foci’ were excluded from data analysis to eliminate potential artefacts from the automatic foci detection algorithm.

### Statistical analysis

Data analysis was performed using SigmaPlot (version 13.0; Systat Software GmbH Erkrath, Germany). The central tendency and variability of data were described by the arithmetic mean and the standard deviation (SD), the distribution of data was represented by box-and-whisker plots. The Wilcoxon signed-rank and Mann-Whitney tests were run to test for significant differences between two groups of paired and unpaired samples, respectively. For three or more groups, for comparisons with repeated measures within study arms the Friedman test was used, for comparisons without repeated measures across study arms the Kruskal-Wallis test. In case of a significant result, Tukey’s or Dunns’s post hoc tests, respectively, were used to pairwise identify groups with significant differences. To test the null hypothesis of equal variances between groups, the robust Brown-Forsythe test was applied. All tests were performed as two-sided tests and *p* values of less than 0.05 were considered as significant. To quantify the effect of different exposures, excess rates, defined as difference between post- and pre-exposure rates, were computed.

## Results

In total, 32 volunteers were included in the study. Their allocation to the three study arms as well as the resulting group characteristics are summarised in Table 1.

**Table 1:** Allocation of 32 volunteers to the three study arms and group-specific characteristics (mean ± SD; range)

Study arm	Sex M/F	Age (years)	Body weight (kg)	BMI (kg/m^2^)	[^18^F]FDG activity (MBq)
SA1	5/5	26.0 ± 3.1; 22–32	77.0 ± 11.3 ^&^; 63–100	25.2 ± 3.8 ^&^; 20.3–33.4	338 ± 42; 287–406
SA2	4/8	25.1 ± 2.2; 23–30	64.7 ± 9.5^&^; 46–80	21.5 ± 2.0 ^&^; 18.0–24.7	
SA3	5/5	24.8 ± 2.7; 21–30	72.9 ± 9.7; 59–87	23.7 ± 2.5; 19.5–28.1	314 ± 43; 259–407

^&^Significant differences between SA1 and SA2 are primarily due to an unbalanced proportion of males and females in SA2

**Fig. 3:**
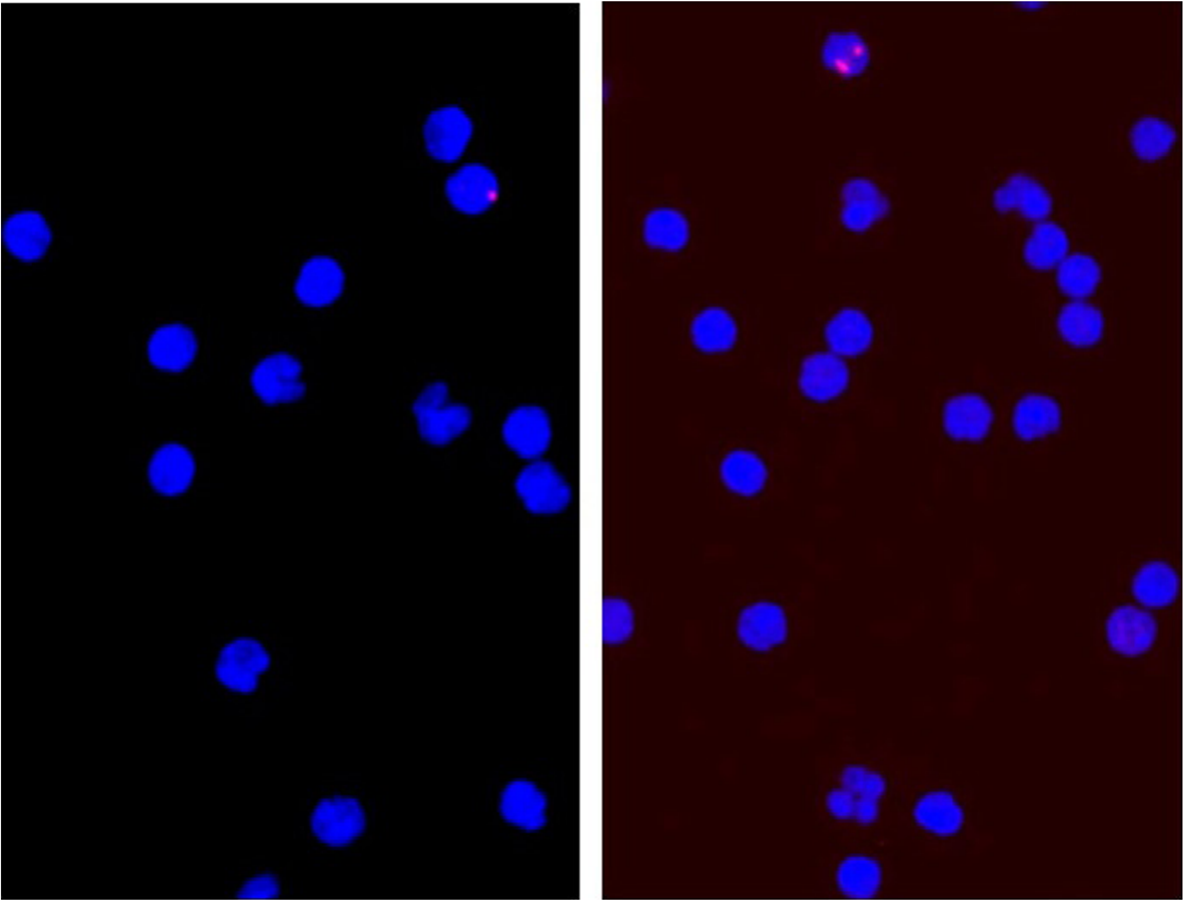
Typical fluorescent microscopy images of lymphocyte nuclei stained in blue showing a nucleus with one (left) and two (right) γH2AX foci stained in red

The volume of blood samples prepared for incubation and postprocessing ranged between 3.0 and 7.5 ml. For each sample, between 1 and 10 (median, 9) replicate slides with at least 1002 (median, 2009) cells per slide were evaluated by the γH2AX-assay. Out of a total of about 4.76 million analysed cells, the percentages of cells with 0, 1, 2, 3, 4, 5, and > 5 foci were 98.58%, 1.292%, 0.093%, 0.020%, 0.007%, 0.004%, and 0.004 %, respectively. On average, 0.016 foci were detected per cell. Pan-nuclear γH2AX staining was observed rarely, but not evaluated. Typical fluorescent images of γH2AX foci in lymphocyte nuclei are shown in Fig. 3.

**Fig. 4:**
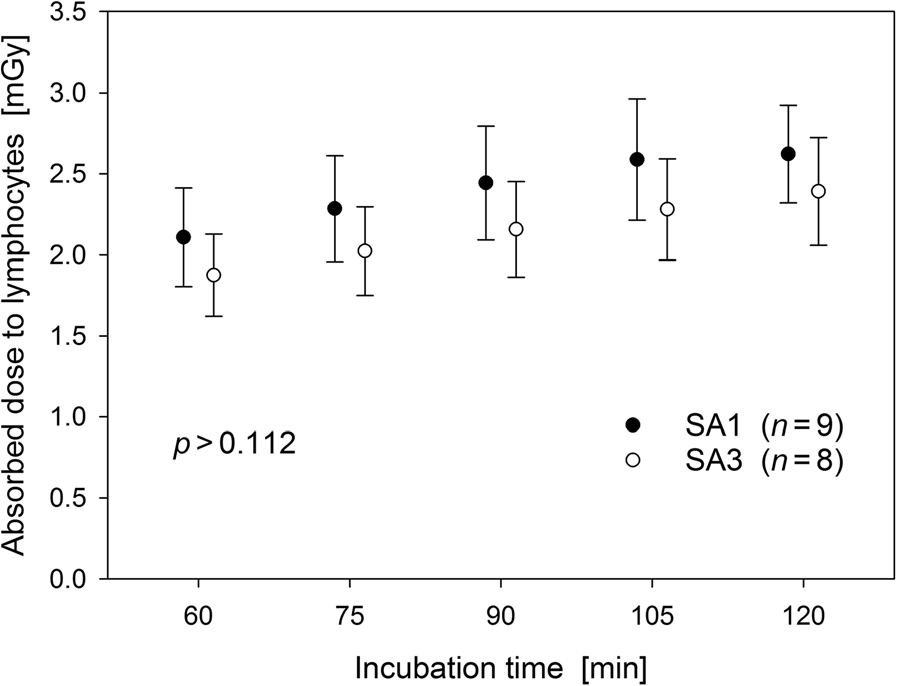
Absorbed doses to blood/lymphocytes exposed to [^18^F]FDG over 60 min in vivo and subsequently up to 60 min in vitro for SA1 and SA3. At each of the five incubation times, doses did not differ significantly from one another (Mann-Whitney test)

Absorbed doses to blood withdrawn 60 min p.i. (SP3) and incubated in vitro over 0, 15, 30, 45, and, 60 min could reliably be estimated for 17 volunteers. Doses ranged between 1.5 and 3.3 mGy. As shown in Fig. 4, the dose increment decreased with time due to the radioactive decay of [^18^F]FDG with a half-life of 110 min. Self-irradiation of blood incubated over 60 min increased the in vivo dose on average by about 25%. For all incubation times, doses differed not significantly between study arms SA1 and SA3 (*p* > 0.112).

**Fig. 5:**
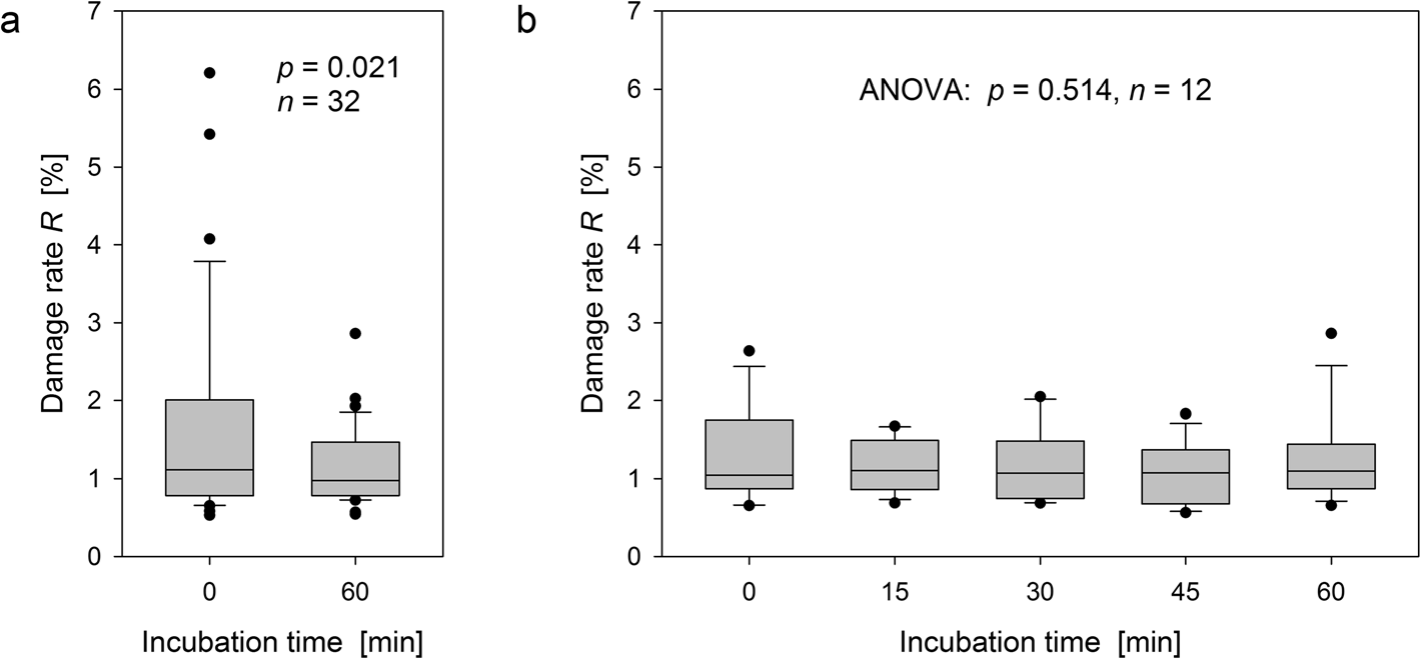
Impact of incubation per se on damage rates of lymphocytes fixed and isolated from blood samples taken (**a**) from all volunteers at sampling point SP1 that were subsequently incubated up to 60 min (Wilcoxon test) and (**b**) from 12 volunteers of SA2 at sampling point SP3 that were subsequently incubated up to 60 min (Friedmann test). The lower and the upper boundary of the boxes indicate the 25th and the 75th percentiles, the solid horizontal lines in the boxes the median, and the whiskers the 10th and the 90th percentiles. Outliers are represented by dots

To determine whether incubation of blood per se altered the γH2AX response, damage rates determined for unexposed lymphocytes fixed either immediately after withdrawal (SP1/IT0) or after subsequent incubation over 60 min (SP1/IT60) were separately pooled over the three study arms. Statistical evaluation of the data in Fig. 5a yielded significantly higher excess damage rates (median Δ*R* = 0.16%, *p* = 0.021) and larger variances (variance ratio = 7.56, *p* = 0.024) for blood immediately fixed after withdrawal. This is presumably an artefact from venipuncture, so that the data for IT0 were disregarded from further evaluations. Under the assumption that the SMF alone has no or only a negligible impact on the γH2AX response (see below), damage rates determined for lymphocytes taken from volunteers of SA2 at SP3 that were incubated over different times provide supplementary information. These rates do not indicate any effect of incubation (Fig. 5b, *p* = 0.514). Moreover, they are congruent with the background damage rates determined for all volunteers at SP1 after incubation of blood over 60 min (Fig. 5a, right). The median value of these comparable background rates was 1.04%.

**Fig. 6:**
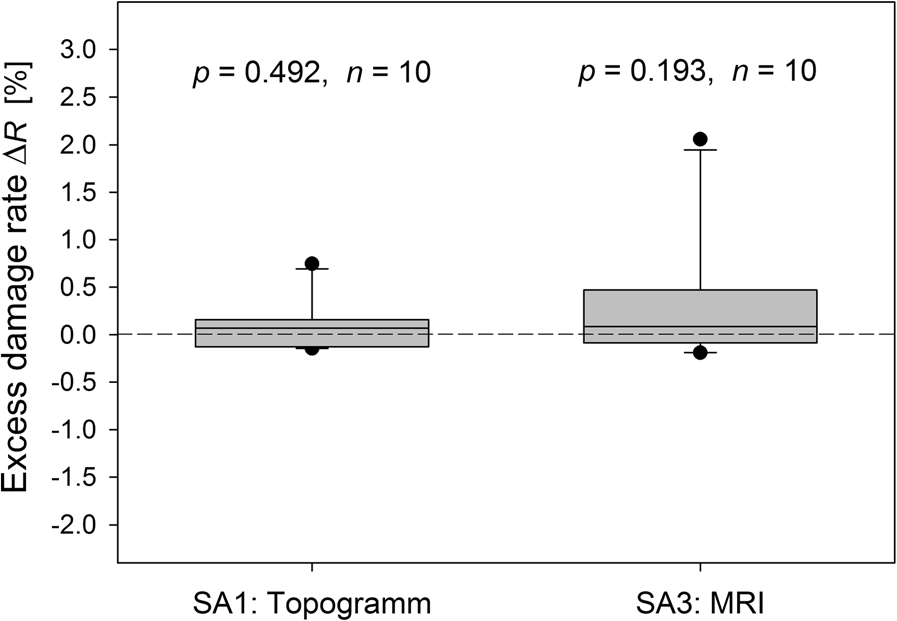
Excess damage rates determined for lymphocytes isolated from blood samples taken before (SP1/IT60) and after (SP2/IT60) the acquisition of the topogram in SA1 and the MRI sequence in SA3 (Wilcoxon test). Scaling is identical with Figs. 7 and 8

As Fig. 6 reveals, neither the X-ray topogram in SA1 (Δ*R* = 0.07%, *p =* 0.492) nor the MRI sequence in SA3 (Δ*R* = 0.09%, *p* = 0.193) affect significantly the damage rates after the respective imaging component (SP2/IT60) compared to the background (SP1/IT60).

**Fig. 7:**
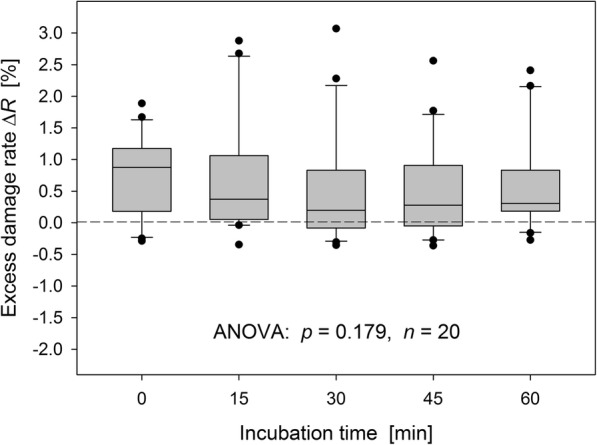
Excess damage rates determined for lymphocytes fixed and isolated from blood samples withdrawn 60 min post [^18^F]FDG injection (SP3) that were subsequently incubated up to 60 min (Friedman test). Data were pooled over SA1 and SA3

Excess damage rates pooled for SA1 and SA3 under the assumption that the SMF did not significantly affect damage rates (see below), are plotted in Fig. 7 for the five considered incubation times. Differences between the groups were not significant (*p* = 0.179). Because incubation per se did not have an effect on damage rates, it is implied that there is approximately a steady state over the considered time range between DSBs caused by the residual [^18^F]FDG in the blood samples and the γH2AX response (cf. the kinetics shown in Fig. 1). Excess rates determined at an incubation time of 60 min can be considered as representative. For these data, the median absolute and relative excess was Δ*R* = 0.31% and Δ*R*/*R*_background_ = 28%, respectively.

**Fig. 8:**
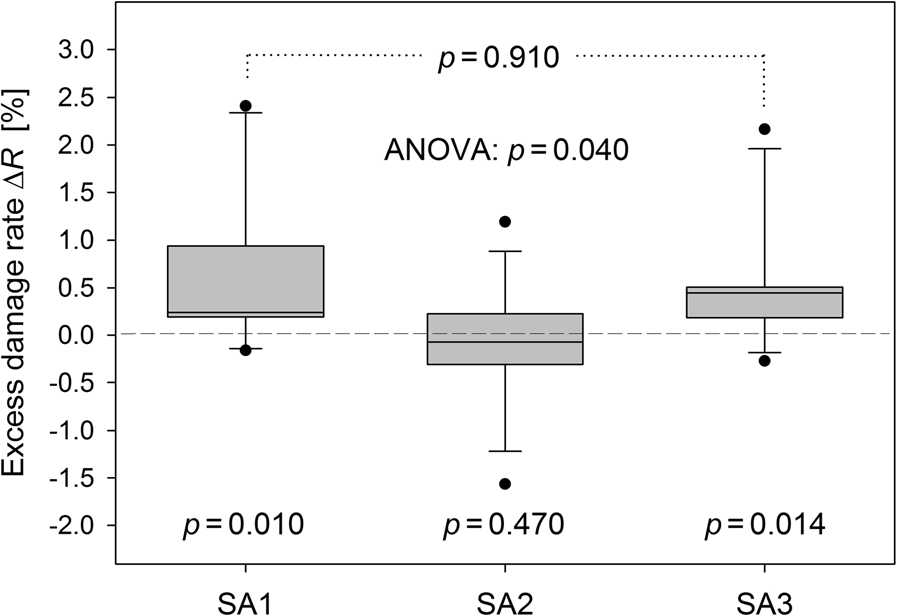
Excess damage rates determined for lymphocytes fixed and isolated from blood samples taken before (SP1/IT60) and after (SP3/IT60) exposure of volunteers in the three study arms. (Excess per study arm, Wilcoxon test; overall differences between study arms, Kruskal-Wallis test; differences between SA1 and SA3, Mann-Whitney test)

Analyses of exposure-related excess damage rates within and between study arms were performed for lymphocytes incubated over 60 min. Excess damage rates (SP3/IT60 vs. SP1/IT60) are shown in Fig. 8 for the study arms. Changes were significant for SA1 (median Δ*R* = 0.24%, *p* = 0.010) and SA3 (median Δ*R* = 0.45%, *p* = 0.014), but not for SA2 (median Δ*R* = − 0.07%, *p* = 0.470). This means that [^18^F]FDG alone and in combination with the SMF has a significant genotoxic effect, in contrast to the SMF alone. A Kruskal-Wallis test yielded a significant overall difference in the excess rates between the study arms (*p* = 0.040). To test for a synergistic or antagonistic effect of the combined exposure of lymphocytes to [^18^F]FDG and the SMF, excess rates of SA1 were compared to SA3, resulting in a non-significant outcome (*p* = 0.910). Qualitatively comparable results were obtained in accordance with the data presented in Fig. 6, when using SP2/IT60 instead of SP1/IT60 as benchmark for study arms SA1 and SA3.

## Discussion

Detrimental health effects of ionising radiation, particularly the induction of cancer becoming clinically manifest only after a latency period of many years, have been demonstrated for large cohorts of exposed persons by long-term follow-up studies. However, radio-epidemiological studies are basically not able to assess health risks associated with new technologies, such as PET/MRI exposing patients simultaneously to two physical agents, at an early stage. In contrast, the γH2AX assay allows to directly monitor DSBs in human DNA caused by genotoxic agents under controlled experimental conditions. Both approaches are linked via the frequency of (misrepaired) radiation-induced DSBs as a prognostic molecular indicator for stochastic health risks in the long run.

In this study, young healthy individuals were enrolled to reduce age- and disease-specific inter-individual variations. This is justified from an ethical perspective because study results will allow a more resilient risk assessment for all and, in particular, young future patients undergoing PET/MRI examinations and the low radiation risk to the study participants [12]. Volunteers of SA1 and SA3 were well matched with respect to sex, age, body weight/mass index, applied activity, and absorbed dose to lymphocytes, which is essential for reliable comparisons between these study arms. In contrast, the proportion of males and females in SA2 was unbalanced, which results in a lower mean body weight/mass index as compared to SA1 and SA3. This is irrelevant, however, because the SMF is modified by the volunteer’s statue only to an entirely negligible extent.

To set-up an appropriate protocol to evaluate DSBs caused by [^18^F]FDG-PET/MRI examinations, two opposite aspects were considered: (i) the steady rise of both the absorbed dose to lymphocytes and the related number of radiation-induced DSBs and (ii) the relatively fast kinetics of DNA repair monitored by the γH2AX assay. Under the reasonable assumption of linear pharmacokinetic processes, at least in the low-dose range, the time-course of γH2AX foci after administration of a radiotracer is mathematically given by a convolution of the time-courses of the dose-rate and the γH2AX response to an instantaneous irradiation, as is approximately given by a short-term X-ray exposure. This relationship was used to compute—for the purpose of illustration—the kinetics shown in Fig. 1. Unfortunately, rate constants for the formation and dephosphorylation of γH2AX foci initiated by a quasi-instantaneous irradiation are not reported and even the given descriptive characteristics of measured response curves are not conclusive. For example, reported times after which the maximum of the γH2AX response is reached following short-term external irradiation vary between 5 min and 1.5 h [16, 18, 38]. Due to this uncertainty and the objective to expose volunteers sufficiently long instantaneously to both agents, volunteers were exposed in vivo over 60 min in the imaging systems and blood samples afterwards additionally in vitro up to 60 min. In this context, it should be recalled that some studies showed a continuing influence of the SMF on the genotoxicity of ionising radiation beyond the end of field exposure [17]. Considering all these aspects and the challenging conditions given in PET/MRI, the employed protocol enabled as much flexibility as possible.

In contrast to previous studies [18, 19], absorbed doses to blood delivered by self- and cross-irradiation were estimated as the most adequate surrogate to quantify the irradiation of lymphocytes by using individual concentration-time courses measured in blood and Monte Carlo computations. Although the activity of [^18^F]FDG administered to the volunteers were quite well adapted to their body weight, blood doses varied considerably within the different exposure groups (Fig. 4). This is primarily attributable to inter-individual variations in the distribution and elimination of the radiotracer from blood and unavoidably contributes to the differences between the evaluated radiation-induced excess damage rates.

Microscope slides were evaluated automatically to score γH2AX foci in a consistent manner and to handle a much larger number of replicate slides, which is indispensable for comparisons in the low dose range, as can be done manually by an experienced technician. Nevertheless, system parameters, such as thresholds for focus size and intensity, must be fixed by the operator and thus can lead to intra- and inter-laboratory variations [17]. To reduce the number of doubtful upward outliers to some extent, the fraction of lymphocytes with at least one focus was evaluated in this study (binary evaluation) instead of the mean number of foci per lymphocyte used in previous studies.

The sensitivity and reliability of the used γH2AX assay, including the implemented automatic foci detection and scoring algorithms, is inter alia directly validated for the specific exposure scenarios investigated in the present volunteer study by the consistent and statistically significant results presented in Fig. 8. In line with published PET studies [18, 19], the genotoxic potential of [^18^F]FDG was statistically verified for SA1 and SA3 comprising different volunteers. Between 60 and 120 min p.i., the radiotracer resulted in a median relative increase of 28% in the rate of lymphocytes with at least one γH2AX focus relative to the background rate, comparable with findings of Schnarr et al. [19]. The data presented by May et al. [18] showed a surprisingly rapid γH2AX response yielding roughly a doubling of the background foci rate in just 5 min p.i., which contrasts strikingly with the almost negligible dose delivered by [^18^F]FDG to blood/lymphocytes over this short period, despite of the high dose rate (as shown in Fig. 1). In any case, these studies as well as our findings prove that the sensitivity of the γH2AX assay is high enough to detect a potential synergism (or antagonism) of the considered effect size between [^18^F]FDG and a strong SMF. However, statistical analysis did not provide any evidence for a genotoxic effect of the SMF, neither alone nor in combination with the radiotracer.

Regarding the genotoxicity of MRI alone, the current scientific evidence is inconsistent. Lancellotti et al. reported that cardiac imaging of healthy men at a 1.5-T MRI system is associated with minor but significant DNA damage [20]. This could, however, not be replicated for humans being exposed in MRI systems operated at 1.5 or 3 T [22–24]. In all these studies, individuals were exposed to the three (electro)magnetic fields generated in combination by a single, arbitrarily chosen MRI sequence so that in case of a positive finding it would not have been possible to identify the causative field. In contrast, Reddig et al. showed that in vitro exposure of human lymphocytes to a 7-T SMF in isolation and also combined with varying magnetic gradient and pulsed radiofrequency fields do not induce DSBs [21]. Principally, it is advisable to investigate the genotoxic potential of the three different fields (alone and in combination with ionising radiation) by a stepwise approach to identify the potentially biological effective field(s).

There are basically three limitations of the present study: (i) the small number of volunteers enrolled. Since healthy young volunteers were exposed to [^18^F]FDG in study arms 1 and 3, it was ethically imperative to limit their number to the absolute necessary to test the stated null hypothesis. (ii) Due to the inaccessibility of the volunteers exposed over 60 min in the PET/MRI system whilst emission data were continuously acquired, it was not possible to withdraw blood during this period, as May et al. did in a PET/CT study [18]. Therefore, the kinetics of the γH2AX response during the first hour p.i. could not be investigated. (iii) The large inter- and intra-individual variation of measured damage rates that limits the detection of minor exposure effects. To overcome this problem, much more volunteers and replication slides would need to be examined, which is hardly feasible.

## Conclusion

The study clearly demonstrated the genotoxic potential of [^18^F]FDG but not of the SMF on human lymphocyte DNA. There was also no indication for a synergism between both physical agents that may be of relevance for risk assessment of patients in PET/MRI. The study was not designed to exclude potential synergistic effects by the magnetic gradient and radiofrequency fields additionally applied in MRI on the genotoxicity of [^18^F]FDG. It forms, however, the essential basis to systematically address this issue separately for both of these fields in further investigations—despite of the ever-present but, as proven in this study, not genotoxic SMF.

## Data Availability

The datasets used and/or analysed during the current study are available from the corresponding author on reasonable request.

## References

[1] C. Catana C. Principles of simultaneous PET/MR Imaging. Magn Reson Imaging Clin N Am. 2017;25(2):231-243. 10.1016/j.mric.2017.01.002 PMC5385858 28390525

[2] Aiello M, Cavaliere C, Marchitelli R, d'Albore A, De Vita E, Salvatore M (2018). Hybrid PET/MRI methodology. Int Rev Neurobiol..

[3] Mannheim JG, Schmid AM, Schwenck J (2018). PET/MRI hybrid systems. Semin Nucl Med..

[4] Ehman EC, Johnson GB, Villanueva-Meyer JE (2017). PET/MRI: Where might it replace PET/CT?. J Magn Resonance Imaging..

[5] Beyer T, Hacker M, Goh V (2017). PET/MRI-knocking on the doors of the rich and famous. Br J Radiol..

[6] Broski SM, Goenka AH, Kemp BJ, Johnson GB (2018). Clinical PET/MRI: 2018 update. AJR Am J Roentgenol..

[7] Miles KA, Voo SA, Groves AM (2018). Additional clinical value for PET/MRI in oncology: Moving beyond simple diagnosis. J Nucl Med..

[8] Brix G, Lechel U, Glatting G (2005). Radiation exposure of patients undergoing whole-body dual-modality 18F-FDG PET/CT examinations. J Nucl Med..

[9] Brix G, Nekolla EA, Borowski M, Noßke D (2014). Radiation risk and protection of patients in clinical SPECT/CT. Eur J Nucl Med Mol Imaging..

[10] Brix G, Reiser M, Semmler W, Hricak H (2007). Risks and safety issues related to MR examinations. Magnetic resonance tomography.

[11] International Electrotechnical Commission. IEC 60601-2-33 (3.2 edition). Particular requirements for the safety of magnetic resonance equipment for medical diagnosis. 2015.

[12] Brix G, Nekolla EA, Nosske D, Griebel J (2009). Risks and safety aspects related to PET/MR examinations. Eur J Nucl Med Mol Imaging..

[13] Mohajer JK, Nisbet A, Velliou E, Ajaz M, Schettino G (2019). Biological effects of static magnetic field exposure in the context of MR-guided radiotherapy. Br J Radiol..

[14] Bug MU, Gargioni E, Guatelli S (2010). Effect of a magnetic field on the track structure of low-energy electrons: a Monte Carlo study. Eur Phys J D..

[15] Lazarakis P, Bug MU, Gargioni E (2012). Effect of a static magnetic field on nanodosimetric quantities in a DNA volume. Int J Radiat Biol..

[16] Löbrich M, Rief N, Kuhne M (2005). In vivo formation and repair of DNA double-strand breaks after computed tomography examinations. Proc Natl Acad Sci USA..

[17] Shi L, Tashiro S (2018). Estimation of the effects of medical diagnostic radiation exposure based on DNA damage. J Radiat Res.

[18] May MS, Brand M, Wuest W (2012). Induction and repair of DNA double-strand breaks in blood lymphocytes of patients undergoing ^18^F-FDG PET/CT examinations. Eur J Nucl Med Mol Imaging..

[19] Schnarr K, Carter TF, Gillis D (2015). Biological response of positron emission tomography scan exposure and adaptive response in humans. Dose Response.

[20] Lancellotti P, Nchimi A, Delierneux C (2015). Biological effects of cardiac magnetic resonance on human blood cells. Circ Cardiovasc Imaging..

[21] Reddig A, Fatahi M, Friebe B (2015). Analysis of DNA double-strand breaks and cytotoxicity after 7 Tesla magnetic resonance imaging of isolated human lymphocytes. PLoS One..

[22] Critchley WR, Reid A, Morris J (2018). The effect of 1.5 T cardiac magnetic resonance on human circulating leucocytes. Eur Heart J..

[23] Fasshauer M, Krüwel T, Zapf A (2018). Absence of DNA double-strand breaks in human peripheral blood mononuclear cells after 3 Tesla magnetic resonance imaging assessed by γH2AX flow cytometry. Eur Radiol..

[24] Suntharalingam S, Mladenov E, Sarabhai T, et al. Abdominopelvic 1.5-T and 3.0-T MR imaging in healthy volunteers: Relationship to formation of DNA double-strand breaks. Radiology. 2018:529–35. 10.1148/radiol.2018172453 29714683

[25] Kuo LJ, Yang LX (2008). Gamma-H2AX - a novel biomarker for DNA double-strand breaks. In Vivo..

[26] Ivashkevich A, Redon CE, Nakamura AJ, Martin RF, Martin OA (2012). Use of the γ-H2AX assay to monitor DNA damage and repair in translational cancer research. Cancer Lett..

[27] Salimi M, Mozdarani H (2014). γ-H2AX as a protein biomarker for radiation exposure response in ductal carcinoma breast tumors: Experimental evidence and literature review. Int J Radiat Res..

[28] Ji J, Zhang Y, Redon CE, Reinhold WC (2017). Phosphorylated fraction of H2AX as a measurement for DNA damage in cancer cells and potential applications of a novel assay. PLoS One..

[29] Pasqualetti G, Gori G, Blandizzi C, Tacca M (2010). Healthy volunteers and early phases of clinical experimentation. Eur J Clin Pharmacol..

[30] Karlberg AM, Sæther O, Eikenes L, Goa PA (2016). Quantitative comparison of PET performance - Siemens Biograph mCT and mMR. EJNMMI Phys..

[31] Nadler SB, Hidalgo JH, Bloch T (1962). Prediction of blood volume in normal human adults. Surgery..

[32] Bolch WE, Eckerman KF, Sgouros G, Thomas SR (2009). MIRD pamphlet No. 21: a generalized schema for radiopharmaceutical dosimetry--standardization of nomenclature. J Nucl Med..

[33] Hays MT, Segall GM (1999). A mathematical model for the distribution of fluorodeoxyglucose in humans. J Nucl Med..

[34] ICRP Publication 110. Adult reference computational phantoms. Ann ICRP. 2009:39(2). 10.1016/j.icrp.2009.09.001 19897132

[35] Kawrakow I, Rogers DWO (2000). The EGSnrc code system: Monte Carlo simulation of electron and photon transport. Report PIRS–701.

[36] Schlattl H, Zankl M, Petoussi-Henss N (2007). Organ dose conversion coefficients for voxel models of the reference male and female from idealized photon exposures. Phys. Med. Biol..

[37] ICRP Publication 107. Nuclear decay data for dosimetric calculations. Annals of the ICRP. 2008;38(3). 10.1016/j.icrp.2008.10.004 19285593

[38] Andrievski A, Wilkins RC (2009). The response of gamma-H2AX in human lymphocytes and lymphocytes subsets measured in whole blood cultures. Int J Radiat Biol..

